# Absence of SMARCB1 in rhabdoid tumor cells increases sensitivity to translation inhibition and alters translation efficiency of specific mRNAs

**DOI:** 10.1016/j.jbc.2024.107988

**Published:** 2024-11-13

**Authors:** Linh T. Nguyen, Anastasia E. Hains, Mohammad O. Aziz-Zanjani, Mattia Dalsass, Sheikh B.U.D. Farooqee, Yingzhou Lu, Peter K. Jackson, Capucine Van Rechem

**Affiliations:** 1 Department of Pathology, Stanford University, Stanford, California, USA; 2 Department of Microbiology & Immunology, Stanford University, Stanford, California, USA; 3 Baxter Laboratory for Stem Cell Biology, Stanford University, Stanford, California, USA; 4 Immagina Biotechnology S.r.l., Pergine Valsugana, Trento, Italy

**Keywords:** cancer biology, chromatin remodeling, translation regulation, mRNA, posttranscriptional regulation, ribosome, SWI/SNF, SMARCB1, rhabdoid tumors

## Abstract

Rhabdoid tumors, characterized and driven by the loss of the mammalian SWItch/sucrose nonfermentable subunit SMARCB1, are very aggressive childhood cancers that can arise in the brain, the kidney, or soft tissues. Cell lines derived from these tumors are specifically sensitivity to the translation inhibitor homoharringtonine. Having recently demonstrated mammalian SWItch/sucrose nonfermentable roles in translation, we assessed SMARCB1 potential roles in translation in rhabdoid tumor cells. We first revealed by cell viability assays that rhabdoid tumor cells’ sensitivity to homoharringtonine were dependent on the absence of SMARCB1. Polysome profiling and immunoprecipitation experiments demonstrated the interaction of SMARCB1 with translation machinery. Global translation assays and ribosome profiling experiments further revealed that SMARCB1 re-expression increased global translation and altered translation efficiency of specific mRNAs. Most regulated mRNAs presented an increased translation efficiency and were involved in differentiation. In comparison with the entire transcriptome, these mRNAs presented a longer coding sequence and were enriched in GC. Finally, we demonstrated that SMARCB1 re-expression increased cytoplasmic localization of these mRNAs and that gene encoding these transcripts were bound by SMARCA4 and SMARCC1. In conclusion, this study reveals that the loss of SMARCB1 in rhabdoid tumors has specific consequences on mRNAs translation with potential to unveil new dependencies.

Rhabdoid tumors are highly aggressive malignancies that can arise in the brain (referred to as atypical teratoid rhabdoid tumors), kidney (referred to as malignant rhabdoid tumors), or soft tissues (1). Rhabdoid tumors typically develop in patients under 3 years of age and often present less than 1-year survival after diagnosis. To date, there is no defined standard of care for these aggressive tumors, and treatments involve a combination of surgery, radiation, and chemotherapy (2).

These childhood cancers have a low mutational rate, and all cases of rhabdoid tumors present mutations in mSWI/SNF (mammalian SWItch/sucrose nonfermentable) subunits: more than 95% in *SMARCB1* and the remaining in *SMARCA4* (3, 4, 5, 6). Within these tumors, *SMARCB1* acts as a tumor suppressor and presents loss of function of both alleles, resulting in the loss of expression of this mSWI/SNF subunit defining these tumors (7, 8, 9).

SMARCB1 is also lost in a variety of other pediatric cancers, arising in the same organs as rhabdoid tumors, such as cribriform neuroepithelial tumors in the brain or renal medullary carcinoma in the kidney, or arising in other locations such as synovial sarcomas (10). In addition, *SMARCB1* is mutated in intellectual disability disorders such as Coffin–Siris syndrome and Kleefstra’s syndrome, indicating alterations of SMARCB1 have devastating pathological outcomes in many contexts (11, 12).

SMARCB1 is a core subunit of two mSWI/SNF complexes: BRG1/BRM-associated factors (BAF) and polybromo-associated BAF complexes (13). SMARCB1 is required for the integrity of BAF and polybromo-associated BAF complexes and is integrated in these complexes during the initial assembly steps, finalizing assembly of the core (13, 14, 15). Structurally, SMARCB1 opposes SMARCA4 in a clamp directly interacting with the nucleosome (16).

mSWI/SNF complexes are evolutionary conserved ATP-dependent remodelers that regulate chromatin accessibility and nucleosome positioning and thus influence gene expression (17, 18). In rhabdoid cells, SMARCB1 loss alters mSWI/SNF targeting at bivalent promoters and enhancers while maintaining specific complexes at superenhancers that are essential for tumorigenesis (14, 15).

Expanding from mSWI/SNF nuclear roles, we recently highlighted the relevance of translation in mSWI/SNF-altered diseases (19). We demonstrated that cancer cells harboring mutations in mSWI/SNF subunits were specifically sensitive to translation pathway inhibitors and presented genetic dependencies with genes encoding translation factors (19). mSWI/SNF subunits localized in the cytoplasm and interacted with the translation machinery (19). Inhibition, depletion, and mutations of mSWI/SNF subunits decreased translation and sensitized cells to translation pathway inhibitors in cell lines of different origins (19).

A screen of small molecules in rhabdoid tumor cell lines identified the translation inhibitor homoharringtonine (HHT) as a drug to which all rhabdoid tumor cell lines were selectively sensitive (20). However, whether this sensitivity is due to the absence of SMARCB1 and whether the absence of SMARCB1 alters translation in rhabdoid tumor cells remained to be determined.

## Results

### Re-expression of SMARCB1 decreases rhabdoid tumors cells’ sensitivity to HHT

To assess the relevance of a role for SMARCB1 in translation in rhabdoid tumors, we used four cell lines engineered to re-express *SMARCB1* (21). These cell lines, derived from rhabdoid tumors of different origin (brain, BT12 and BT16; kidney, G401; and liver, TTC549), were modified to conditionally express *SMARCB1* in the presence of doxycycline (14, 21). Control cell lines express *GFP* under the same “TET on” system. However, the cell of origin of rhabdoid tumor being unknown, we could not directly compare re-expressed levels of SMARCB1 to original levels. We demonstrate SMARCB1 re-expression levels to be lower than endogenous levels in neural precursor cells which, as rhabdoid tumor cells, have the same origin (neural crest) (22, 23), and are not fully differentiated (Fig. S1*A*).

**Figure 1:**
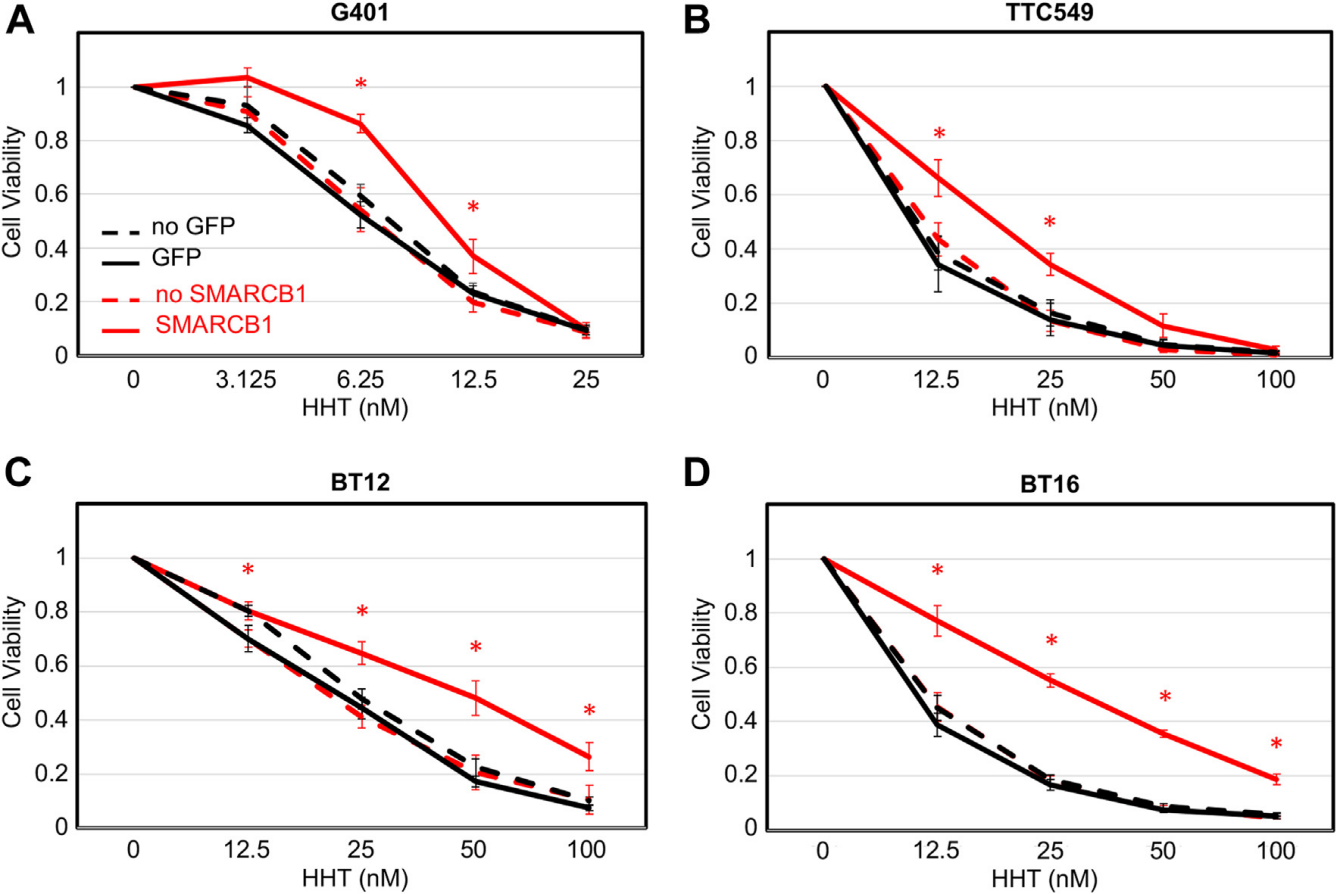
**Re-expression of SMARCB1 in rhabdoid tumor cells decreases sensitivity to the inhibitor of translation homoharringtonine.** Cell viability assays in the presence of the translation inhibitor homoharringtonine (HHT) at indicated concentrations for 72 h (CellTiter-Glo 2.0 Cell Viability Assay, Promega) in G401 (A), TTC549 (B), BT12 (C), and BT16 (D) after 24 h of doxycycline induction of SMARCB1 (*red*) or GFP (*black*). Cell viability was calculated as a fraction of the control (dimethylsulfoxide = 0 nM) viability. Averages of at least three experiments are represented. Statistics: Student’s *t* test: ∗*p* < 0.05 related to no SMARCB1.

We demonstrate that re-expression of SMARCB1 increases rhabdoid tumor cells’ viability in the presence of a range of concentrations of HHT (Fig. S1, *B–E* and Fig. 1, *A–D*, red lines), while expression of GFP does not (Fig. 1, *A*–*D*, black lines). However, re-expression of SMARCB1 does not alter sensitivity to the mammalian target of rapamycin inhibitor AZD8055 (Fig. S1, *F–I*).

### Re-expression of SMARCB1 increases mSWI/SNF subunits’ presence with the translation initiation machinery

**Figure 2:**
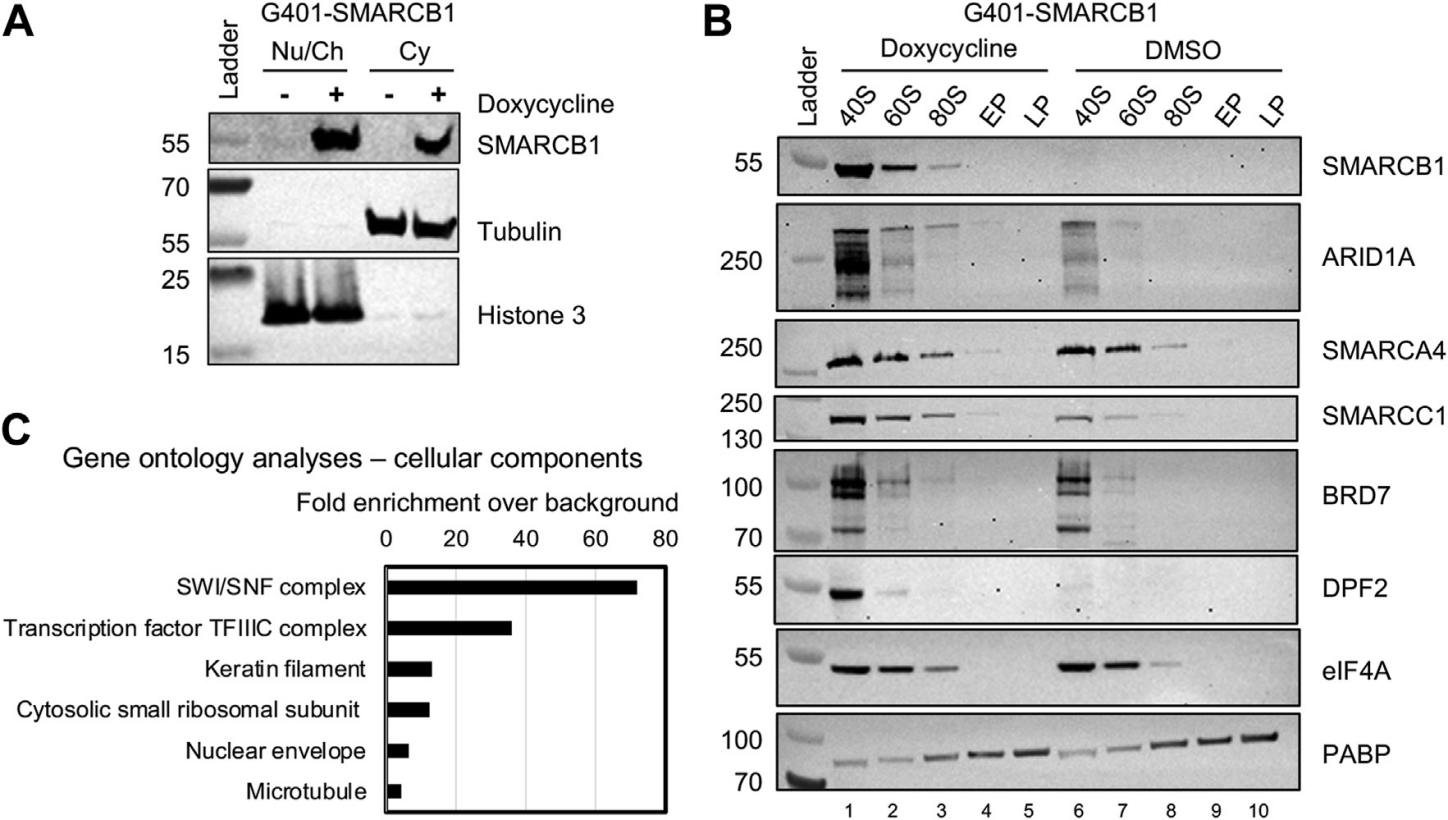
**Cytoplasmic SMARCB1 interacts with other mSWI/SNF subunits and with the translation machinery.***A,* upon re-expression, SMARCB1 localizes in the nucleus and the cytoplasm of G401 cells. Nu/Ch = nucleus and chromatin combined. *B,* SMARCB1 cosediments with initiating fractions of translation (40S-60S-80S, lanes 1–3), and stabilizes ARID1A, SMARCA4, SMARCC1, BRD7, and DPF2 in the same fractions (compare lanes 1–3 to lanes 6–8). *C,* Gene ontology analyses of proteins identified by mass spectrometry after immunoprecipitation of SMARCB1 from G401 cytoplasmic fraction in 300 mM salt. mSWI/SNF, mammalian SWItch/sucrose nonfermentable; Cy, cytoplasm; EP, early polysome; LP, late polysome.

Having previously demonstrated the presence of mSWI/SNF subunits (including SMARCB1) in the cytoplasm of other cell lines and their interaction with the translation machinery, we wanted to assess whether SMARCB1 would localize in the cytoplasm in rhabdoid tumor cell lines as well. To do so, we performed biochemical fractionation of G401 cells followed by Western blot. We demonstrate that, upon re-expression, SMARCB1 localizes both in the nucleus and the cytoplasm of G401 cells (Fig. 2*A*). Polysome profiling experiments further revealed that SMARCB1 and other tested mSWI/SNF subunit cosediment in initiating fractions of translation (40S, 60S, 80S, Fig. 2*B*). Re-expression of SMARCB1 increased the presence of tested mSWI/SNF subunits in these fractions: ARID1A, SMARCA4, SMARCC1, BRD7, and DPF2 (compare lanes 6–8 = no SMARCB1 to 1–3 = SMARCB1), consistent with mSWI/SNF forming complexes in the cytoplasm. To confirm these interactions, we performed immunoprecipitation experiments from cytoplasmic extracts in 300 mM NaCl followed by mass spectrometry, as performed to assess strongly interacting proteins (13). These experiments confirmed SMARCB1’s cytoplasmic interaction with 15 mSWI/SNF subunits [Fig. 2*C* and Fig. S2*A* (green dots), *B*, and *C*] (24). We further revealed the interaction of SMARCB1 with ribosomal and translation-related proteins [Fig. 2*C* and Fig. S2*A* (magenta dots), *B*, and *D*].

### SMARCB1 increases global translation and alters translation efficiency of specific mRNAs

We then examined the consequences of SMARCB1 re-expression on translation. We first assessed global translation by incorporation of the amino acid analog L-azidohomoalanine (AHA). Re-expression of SMARCB1 increased global translation by 35% (Fig. S3*A*).

Since a global role on translation does not preclude a role on specific mRNAs (25, 26), we wanted to assess whether re-expression of SMARCB1 could alter the translation efficiency of specific mRNAs. To do so, we performed ribosome profiling experiments along total RNA-seq. Ribosome profiling enables to extract ribosomes in active translation and to purify the ribosome protected fragments, hence directly assessing mRNAs being actively translated (27, 28). Coupling ribosome profiling with RNA-seq further enables to compare actively translated mRNAs to their levels and to assess whether translation efficiency is altered under the tested conditions; in other words, altered translation efficiency means that the increase/decrease in translation is not the only consequence of the increase/decrease level of the respective mRNA.

We demonstrate that re-expression of SMARCB1 leads to various consequences on mRNAs: alteration of translation only (Fig. S3*B*, green dots), alteration of RNA levels only (Fig. S3*B*, purple dots), unidirectional alteration of translation and RNA levels (Fig. S3*B*, blue dots). Opposite effects on translation and RNA levels were almost inexistent (Fig. S3*B*, orange dots).

**Figure 3:**
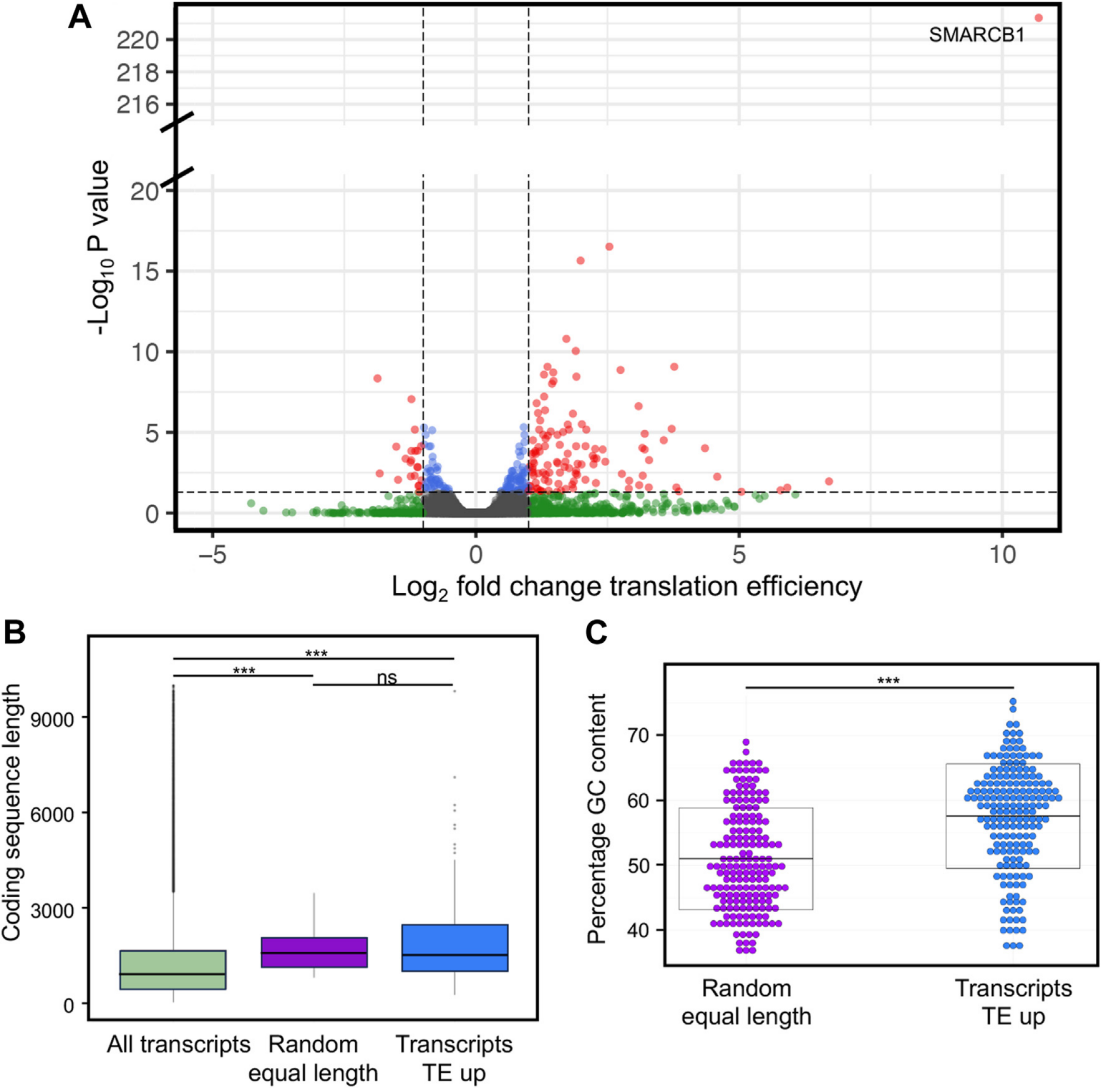
**Re-expression of SMARCB1 alters the translation efficiency of specific mRNAs.***A,* translation efficiency analyses from ribosome profiling and total RNA-seq of G401 cells after 12 h of doxycycline induction of SMARCB1 compared to dimethylsulfoxide. Significant transcripts (adjusted *p* value < 0.05) with a fold change >2 are represented in *red*. *B,* coding sequence length of all transcripts in the genome (*green*), transcripts with increased translation efficiency upon SMARCB1 re-expression (*blue*), and of a random set of transcripts with comparable length of the green transcripts for further analyses (*purple*). *C,* GC content of transcripts with increased translation efficiency upon SMARCB1 re-expression (*blue*) compared to a random set of transcripts of equal length (*purple*). Statistics: Mann–Whitney *U* test was applied for two groups, and *p* values were calculated and adjusted with the Benjamini–Hochberg method. ∗∗∗ adjusted *p* values < 0.001.

By analyzing the ratio of ribosome profiling over RNA-seq, we revealed that re-expression of SMARCB1 increased translation efficiency of 180 mRNAs and decreased translation efficiency of 25 mRNAs (Fig. 3*A*). These results were specific to the re-expression of SMARCB1 because expression of GFP altered translation efficiency of very few nonoverlapping mRNAs (Fig. S3*C*). Gene Ontology analyses of mRNA presenting increased translation efficiency revealed that these mRNAs present enrichments in biological processes related to muscle and cell differentiation and in cellular components related to muscle (Fig. S3*D*).

### SMARCB1 increases translation efficiency of long and GC-rich mRNAs

When compared to all transcripts in the genome, the 180 transcripts presenting increased translation efficiency upon SMARCB1 re-expression had a significantly longer coding sequence length (Fig. 3*B*, compare red to green boxes). The 25 transcripts presenting decreased translation were also significantly longer (Fig. S3*E*, compare red to green boxes). We further analyzed the codon usage by comparing these 180 transcripts to a random set of transcripts of the same size (Fig. 3*C* and Fig. S3*F*, blue boxes). Our analyses of the ribosome footprints of transcripts with increased translation efficiency revealed 46/64 codons with differential usage with adjusted *p* value <0.05, among which 38/64 codons presented an adjusted *p* value <0.01. Further analyses revealed that 68% of GC content belonged to significant codons. GC content analyses of mRNAs with increased translation efficiency upon SMARCB1 re-expression confirmed these transcripts to be enriched in GC (Fig. 3*C*). This was specific to transcripts with increased translation efficiency because transcripts presenting decreased translation efficiency were not enriched in GC (Fig. S3*F*).

### SMARCB1 increases cytoplasmic localization of mRNAs with higher translation efficiency

**Figure 4:**
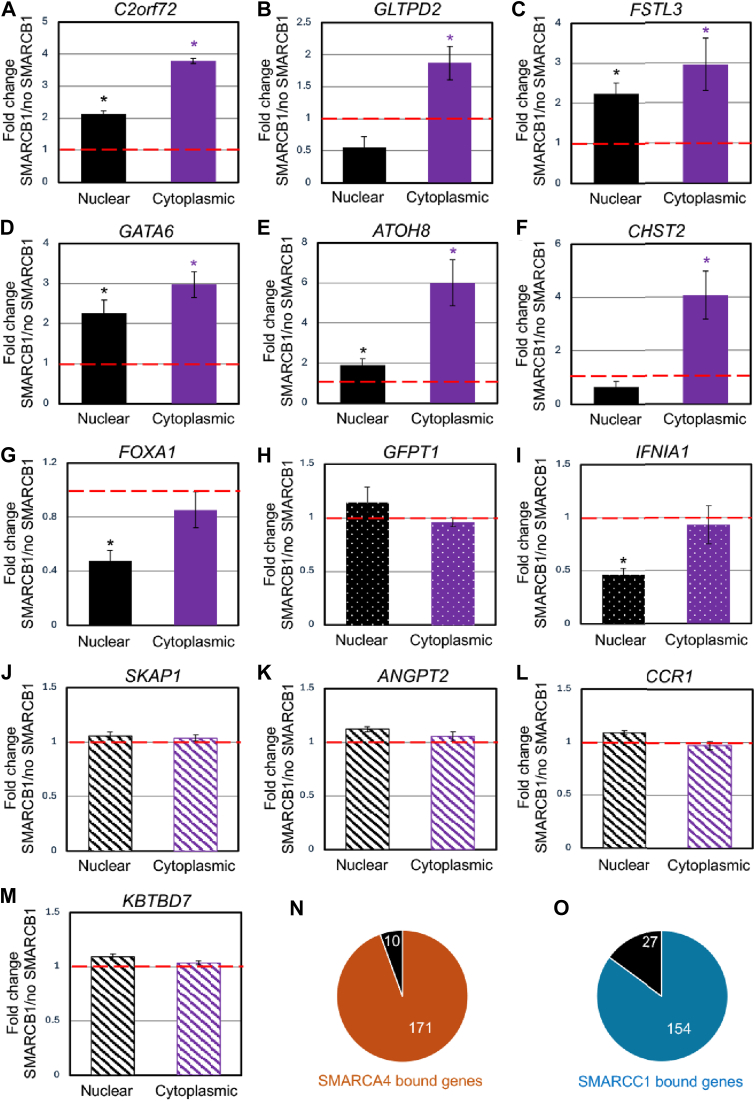
**Re-expression of SMARCB1 increases the cytoplasmic localization of transcripts presenting increased translation efficiency.***A-M,* fold change of annotated transcripts in the nucleus and the cytoplasm after 24 h of SMARCB1 induction with doxycycline compared to dimethylsulfoxide, relative to 18S. *Full bars* are transcripts with increased translation efficiency upon SMARCB1 induction, *dotted bars* are transcripts from the random set of transcripts of similar length, and *stripped bars* are transcripts with decreased translation efficiency upon SMARCB1 induction. Statistics: Student’s *t* test: ∗*p* < 0.05 related to no SMARCB1. *N-O,* proportion of genes encoding the transcripts with increased translation efficiency upon SMARCB1 re-expression bound by SMARCA4 (J) or SMARCC1 (K).

Because high GC content in mRNAs was shown to facilitate their cytoplasmic localization and enhance their translation (29), we tested whether SMARCB1 would facilitate the cytoplasmic localization of these mRNAs by assessing their subcellular localization in G401 cells with and without re-expression of SMARCB1. We demonstrate that SMARCB1 re-expression increases the cytoplasmic localization of six out of seven tested mRNAs with increased translation efficiency (Fig. 4, *A*–*G* and Fig. S4*A and B*), while expression of GFP did not (Fig. S4, *C–E*). We further tested two mRNAs from our random set of transcripts of the same size and demonstrate these mRNAs did not present cytoplasmic enrichment upon SMARCB1 re-expression (Fig. 4, *H* and *I* and Fig. S4, *J–K*). Demonstrating specificity for mRNAs with increased translation efficiency, SMARCB1 re-expression did not alter the subcellular localization of mRNAs presenting decreased translation efficiency (Fig. 4, *J–M* and Fig. S4, L–O). We further assessed whether mRNAs with decreased translation efficiency presented altered distribution within polysome profile fractions. Quantitative PCR from 80S, early polysomes (first three polysomes), and late polysomes (from fourth polysome to the end of the profile) demonstrate that mRNAs with decreased translation efficiency were less abundant in all fractions upon SMARCB1 re-expression (Fig. S4, *P–Q*). Furthermore, *C2orf72*, an mRNA presenting increased translation efficiency upon SMARCB1 re-expression, was enriched in all fractions, while small variations in *GAPDH* abundance were not significant (Fig. S4*R*).

We finally wanted to assess whether transcripts with increased translation efficiency could be also controlled by SMARCB1 at the transcriptional level. To do so, we analyzed publicly available chromatin immunoprecipitation (ChIP) sequencing of SMARCA4 and SMARCC1 in G401 cells expressing SMARCB1 (15) and revealed that 94% and 85% of genes encoding transcripts with increased translation efficiency upon SMARCB1 re-expression were bound by SMARCA4 and SMARCC1, respectively (Fig. 4, *J* and *K*). Of note, in absence of SMARCB1 77% and 62% of these genes were still bound by SMARCA4 and SMARCC1, respectively. These observations are consistent with published data reporting that the absence of SMARCB1 in G401 cells lead to mSWI/SNF complexes absence or reduction at target genes (15). Together, these results are consistent with mSWI/SNF controlling transcription, export, and translation of these transcripts.

## Discussion

A challenge with cancers driven by the loss of tumor suppressors is the inability to target the driver of tumorigenesis. Therefore, understanding the consequences of such loss is necessary to unveil potential targeted therapeutic strategies.

Within this study, we reveal that rhabdoid cells’ sensitivity to the transaction inhibitor HHT is reduced upon SMARCB1 re-expression. We further reveal that SMARCB1 re-expression increases global translation. These results are consistent with our previous findings describing decreased global translation upon mSWI/SNF subunits’ absence or inhibition (19). This global decrease of translation could explain the increased sensitivity to translation inhibition, with translation becoming too low for the cells. Whether this decreased translation leads to a decreased cell growth and/or to cell death remains to be determined.

A global role on translation does not preclude a role on specific mRNAs. For example, upon stress, the translation initiation factor eukaryotic initiation factor 2 alpha reduces global translation and preferentially translates specific mRNAs (25). To take another example, the reduced number of ribosomes in Diamond–Blackfan anemia alters polysome profiling globally and the regulation of specific transcripts (26). We demonstrate that SMARCB1 re-expression alters translation efficiency of 205 mRNAs; 180 mRNAs presented increased translation efficiency; and 25 mRNAs presented decreased translation efficiency. Gene ontology analyses of mRNAs with increased translation efficiency revealed biological processes and cellular components linked to muscle and differentiation, consistent with rhabdoid tumors having a neural crest origin and being stalled in development toward a mesenchymal differentiation (22, 23).

**Figure 5:**
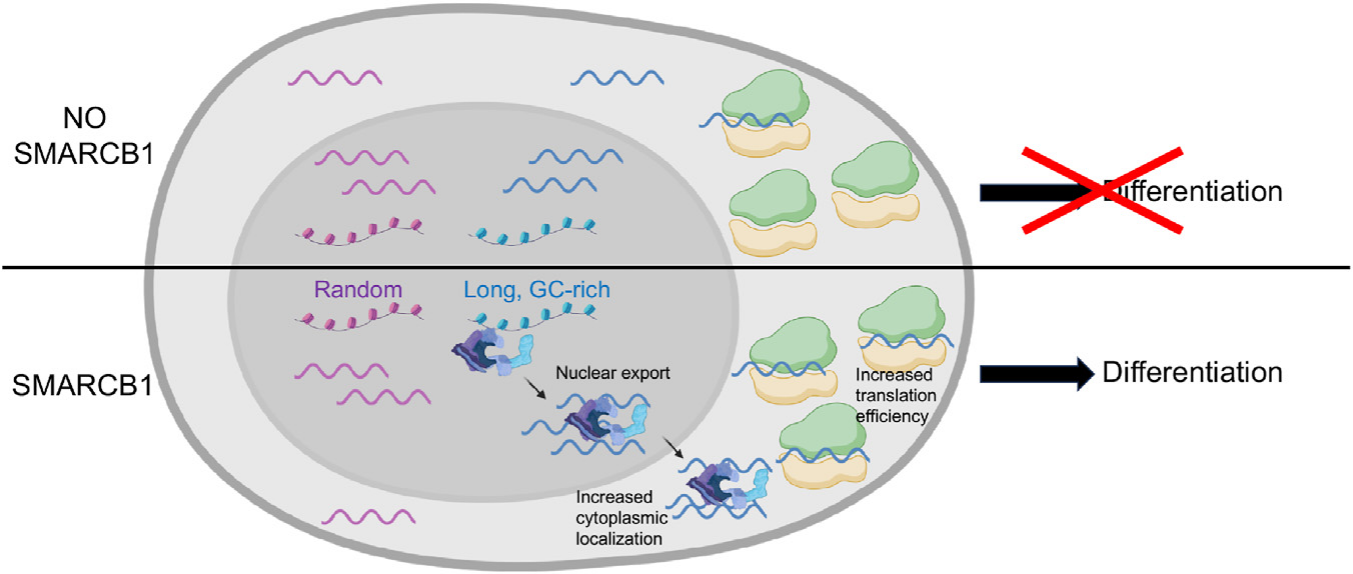
**Model.** SMARCB1, as part of mSWI/SNF complexes, regulates long and GC-rich transcripts from transcription to export and translation, leading to proper cell differentiation. Created in BioRender. Van Rechem, C. (2024) BioRender.com/v32s695. mSWI/SNF, mammalian SWItch/sucrose nonfermentable.

While this study, along our previous work (19), is consistent with direct roles for mSWI/SNF complexes in translation, they do not exclude the potential contribution of nuclear functions to the observed altered translation. In fact, our results suggest that increased translation efficiency of mRNAs by SMARCB1 is accomplished through a combination of nuclear and cytoplasmic roles (Fig. 5). Indeed, the presence of mSWI/SNF subunits at genes encoding these mRNAs and their increased cytoplasmic localization upon SMARCB1 re-expression suggest mSWI/SNF to regulate these transcripts from transcription to export and translation. Such functions have already been reported for other factors. For example, upon stress, the translation elongation factor eukaryotic elongation factor 1A1 localizes in the nucleus where it activates the transcription of a heat shock gene; it then travels with the polymerase along the gene before stabilizing the mRNA and transporting it to the ribosomes where it gets translated (30).

It remains to be determined whether SMARCB1 directly and/or indirectly regulates the translation of transcripts with decreased translation efficiency. The lower abundance of these transcripts in 80S, early, and late polysomes upon SMARCB1 re-expression is consistent with a role for SMARCB1 in initiation of translation. More experiments are needed to mechanistically determine how SMARCB1 induces a decreased translation efficiency of specific transcripts and whether this is direct or not, such as through the transcriptional regulation of a factor regulating the translation of these transcripts.

The mechanism by which SMARCB1 and mSWI/SNF complexes directly regulate translation remains to be determined. In this study, we demonstrate cytoplasmic SMARCB1 to interact with several ribosomal subunits under high salt conditions, consistent with direct/stable interaction. Five out of the six detected ribosomal proteins were also detected after immunoprecipitation of SMARCA4 in other cell types (19), suggesting a shared mechanism within distinct cellular models. Specifically, we consistently detected a high number of RPS3 peptides. RPS3 is at the ribosome entry channel, promoting mRNA binding and stabilizing the preinitiation complexes at start codons (31). RPS2, also enhancing initiation at the entry channel, was also found interacting with SMARCA4 (32). Analysis of the structure of the human 80S ribosome on the Protein Data Bank website (RCSB.org) (33) reveals RPS15A in proximity of RPS2, and RPS13 in proximity of RPS15A; these ribosomal proteins were both detected after immunoprecipitation of SMARCB1 and SMARCA4. These observations, along with the results reported in this study, suggest the mSWI/SNF complexes to bring mRNA directly to the ribosome entry channel to help stabilize and initiate translation. This possibility is consistent with our detection of mSWI/SNF subunits in initiating fractions of polysome profiles.

In conclusion, this study reveals that the loss of SMARCB1 in rhabdoid tumors has specific consequences on mRNAs translation with potential to unveil new dependencies and future therapeutic strategies.

## Experimental procedures

### Cell culture

Rhabdoid tumor cell lines were obtained from Dr Roberts’ laboratory (St Jude Children’s Research Hospital). Cells were cultivated in McCoy’s 5A (Sigma-Aldrich 8403) supplemented with 10% fetal bovine serum (Biowest S162TA), 1% P/S and 1% Glutamax (Thermo Fisher Scientific 35050061). *SMARCB1* and *GFP* were induced with 0.1 μg/ml doxycycline (Sigma-Aldrich D3072-1ML) for the indicated period of times.

### Cell viability assays

Cells were seeded at 1 × 10^3^ density in triplicate in 96-well plates. After 24 h, cells were treated with 0.1 μg/ml doxycycline for 24 h before being treated with HHT (Tocris 1416) or AZD8055 (Achemblock 10280) for 72 h. Viability assays were performed following supplier’s instructions from the CellTiter-Glo 2.0 Cell Viability Assay (Promega). Luminescence reading was performed using GloMax Explorer Microplate Reader (Promega).

### Biochemical fractionations

#### Protein analyses

Cell fractionations were performed as in (34). For the Western blot analysis a comparable fraction of each compartment was loaded on a gel.

#### RNA analyses

RNAs from the nucleus and cytosol were extracted with the RNA Subcellular Isolation Kit (Active Motif, 25501) following supplier’s instructions.

### Antibodies

SMARCA4 (Cell Signaling 52251S), SMARCB1 (Santa Cruz Biotech 166165), ARID1A (Cell Signaling 12354S), SMARCC1 (Abcam ab172638), DPF2 (Abcam ab134942), PABP (Abcam ab21060), eIF4a1 (Abcam 31217), V5 tag (Thermo Fisher Scientific R960-25), TUBULIN (Santa Cruz Biotech sc-32293), histone H3 (Abcam ab1791), Strep-HRP (Invitrogen SA10001), GAPDH (Abcam ab9485), BRD7 (Protein tech 51009-2-AP), IgG2a isotype control (Cell Signaling 61656S).

### Global translation assay

AHA incorporation was performed as in (35) following the supplier's instructions from Click-IT Metabolic Labeling Reagents for Proteins (Life Technologies); AHA was added for the last 2 h of doxycycline treatment (24 h). Antibodies intensities from Western blot membranes were analyzed using ImageJ (https://imagej.net/ij/). Because of the string smear obtained for these Western blots, loading controls (GAPDH) were ran on separate membranes. Quantification panel represents the average of six independent experiments.

### Polysome profiling

Polysome profiling was performed as in (35). Because of the high number of proteins tested and size restriction, fractions from polysome profiles were assessed with several gels. For quantitative polymrease chain reaction (qPCR) analyses, RNAs from 500 μl of each fraction (80S, early polysomes, late polysome) were purified by Trizol extraction; two replicates were analyzed.

### Ribosome profiling

Cells were seeded at 1.5 × 10^6^ cell in 15-cm plates. After 24 h, cells were treated with 0.1 μg/ml doxycycline or dimethylsulfoxide (DMSO) for 12 h. Six plates per condition were harvested and pooled together. Ribosome profiling was performed using the RiboLace gel free kit (Immagina Biotechnology) following supplier’s instructions. For global RNA-seq, stranded RNA libraries were prepared using the NEBNext Ultra II Directional RNA Library Prep Kit for Illumina. Experiments were performed in duplicate.

#### Analyses

Reads were aligned to the genome for both RNA-seq and ribosome profiling with STAR Aligner (36). Reads per gene were counted by using count module of HTSeq tool with options “–mode = intersection-strict” for both RNA-seq and ribosome profiling and “—type = exon” for RNA-seq and “—type = CDS” for ribosome profiling. For aligning and counting, GENECODE human annotation v.42 was used. To find differentially expressed genes, the R-package DESeq2 (https://bioconductor.org/packages/release/bioc/html/DESeq2.html) (37) was used and resulting *p* values were adjusted using the Benjamini–Hochberg correction method. For gene length and GC content, Mann–Whitney *U* test was applied for two groups and *p* values were calculated and adjusted with the Benjamini–Hochberg method (38).

### Immunoprecipitation

G401 cells were seeded for 24 h prior treatment with 0.1 μg/ml doxycycline or DMSO for 24 h. Cell pellets were fractionated to isolate cytoplasmic fractions with EB0 [50 mM Tris–HCl pH 7.5, 0.1% NP-40, 1 mM EDTA, 1 mM MgCl2, 1x protease and phosphatase inhibitor (Thermo Fisher Scientific 78446)], and nuclear fractions with EB300 (50 mM Tris–HCl pH 7.5, 300 mM NaCl, 1% NP-40, 1 mM EDTA, 1 mM MgCl2, 1x protease, and phosphatase inhibitor). Fractions were re-equilibrated to equal salt concentration (300 mM NaCl). One milligram of protein from each fraction was precleared with Dynabeads protein G (Invitrogen 10004D) for 1 h at 4 °C. Five hundred micrograms of protein of each fraction were incubated with V5 tag or IgG2a antibodies overnight at 4 °C. Twenty five microliters of Dynabeads protein G were added for 10 min at 4 °C, followed by two washes with EB300, and one final wash with EB300 with 0.1% NP-40.

### Mass spectrometry

#### Chemicals and reagents

2-Chloroacetamide (Sigma-Aldrich C0267), potassium hydroxide (Sigma-Aldrich P5958), TFA (Thermo Fisher Scientific AAL06374AC), acetic acid (Thermo Fisher Scientific A11350), Optima LC/MS Grade Water (Thermo Fisher Scientific W6-4), 99.5% formic acid LC/MS Grade (Thermo Fisher Scientific A117-50), tris(2-carboxyethyl)phosphine hydrochloride (Thermo Fisher Scientific PG82080) and Halt Protease and phosphatase inhibitor cocktails, EDTA-Free (Thermo Fisher Scientific 78425 and 78428, respectively), LC/MS grade acetonitrile (Honeywell 14261-1L), Trypsin/Lys-C Mix, Mass Spec Grade (Promega V5073), and Empore C18 47 mm Extraction Disk (Empore 320907D). Liquid chromatography was conducted using a Bruker Pep-Sep C18 10 cm packed column with 1.5 μm beads and 150 μm internal diameter (1893483) attached to a ZDV Sprayer with 20 μm internal diameter (1865710).

#### Sample preparation

Fifty microliters of Trypsin/Lys-C Mix at a concentration of 0.01 μg/μl in 100 mM ammonium bicarbonate was added to the beads and incubated for 30 min at room temperature. After magnetic separation, the supernatant was collected, and the beads were washed with 20 μl of 2 M urea in 100 mM ammonium bicarbonate. Reduction/alkylation buffer was composed of 100 mM tris (2-carboxyethyl) phosphine and 300 mM two-chloroacetamide, with pH adjusted to 7 to 8 using KOH. The pH of the buffer was verified using a pH indicator strip. This buffer was prepared immediately before use to ensure the full activity of 2-chloroacetamide, and 7 μl of it was added to the samples and incubated for 30 min in the dark at room temperature. An additional 50 μl of Trypsin/Lys-C Mix was added for a 3-h incubation at 37 °C. Postdigestion, the samples were acidified with TFA. Stage-Tips were constructed using two C18 Empore disks, washed with methanol and acetonitrile/acetic acid solutions, loaded with digested peptides in 1% acetic acid, and further washed. Finally, the peptides were eluted twice using 30 μl 50% acetonitrile/0.1% acetic acid solution.

#### LC-MS analysis

A nano Elute ultrahigh-pressure nano-flow chromatography system (Bruker) was used with a timsTOF HT mass spectrometer equipped with a CaptiveSpray nano-electrospray ion source. Chromatography was performed on a Pep-Sep C18 column at 50 °C, using a mobile phase consisting of water/acetonitrile**/**formic acid (mobile phase A) and acetonitrile/formic acid (mobile phase B). A 60-min linear gradient from 2 to 33% mobile phase B was employed, followed by a high-percentage B wash and re-equilibration. The flow rate was maintained at 500 nl/min. The mass spectrometer was operated in data dependent analysis-parallel accumulation-serial fragmentation mode, performing four parallel accumulation-serial fragmentation tandem mass spectrometry scans per cycle as described before (39).

#### Data analysis

Raw data files were processed using MS Fragger software (GitHub - Nesvilab/MSFragger: Ultrafast, comprehensive peptide identification for mass spectrometry–based proteomics) against the NCBI *Homo sapiens* RefSeq protein database. Search parameters included collision induced dissociation fragmentation ion types with a precursor error tolerance of 20 ppm and a fragment ion tolerance of 40 ppm. Searches included S/T/Y phosphorylation and up to three modifications per peptide beside standard modifications. Peptides were validated using Percolator and Protein Prophet at 1% false discovery rate. Protein quantification was performed using IonQuant, with normalization across runs and match-between-runs settings accommodating retention time and ion mobility tolerances of 0.4 min and 0.05 (1/K0), respectively. Further analysis was conducted using Frag-Pipe Analyst (40).

### qPCR

RNA was converted to complementary DNA with Super Script IV Reverse Transcriptase (Invitrogen, 18090200). qPCR was performed with Applied Biosystems Power Sybr Green PCR Master Mix (Applied Biosystems, 4368708) with a Quantstudio five Real-Time PCR System (A28140). Primer sequences utilized are reported below. For qPCR from fractionations, ΔCt values were calculated by subtracting the target Ct value with control gene *18S* value. For qPCR from polysome profiles fractions, ΔCt values were calculated by subtracting the Dox-induced SMARCB1 Ct value with the DMSO control. For primers used see supplemental table 1.

### ChIP-sequencing analyses

ChIP sequencing fastq files were obtained from GSE90634 (15). Fastq files were aligned to the hg38 genome with HISAT2 (41) and were further sorted and indexed with samtools (42). MACS2 (43) was used to call peaks from the resulting bam files.

### Quantification and statistical analyses

Unless otherwise annotated in specific experimental procedures subsections, experiments were performed at least three independent times. Unless otherwise annotated in specific experimental procedures subsections, data are expressed as mean ± SEM. *p* values < 0.05 calculated with a two-tailed students’ *t* test were considered significant and annotated with ∗.

## Data availability

The data generated in this study is publicly available in the Gene Expression Omnibus repository under accession number GSE268318. ChIP sequencing fastq files were obtained from GSE90634 (15).

## Supporting information

This article contains supporting information (24).

## Conflict of interest

M. D. is and employee of Immagina Biotechnology. The other authors declare that they have no conflicts of interest with the contents of this article.
