# Supplementary material for: Absence of SMARCB1 in rhabdoid tumor cells increases sensitivity to translation inhibition and alters translation efficiency of specific mRNAs
Source: J Biol Chem. 2024 Nov 13;300(12):107988. doi: 10.1016/j.jbc.2024.107988 (PMC11699736; doi:10.1016/j.jbc.2024.107988)
Supplement: Supplemental Figures [file mmc1.docx]

**Absence of SMARCB1 in rhabdoid tumor cells increases sensitivity to translation inhibition and alters translation efficiency of specific mRNAs.**

Linh T. Nguyen^1^, Anastasia E. Hains^1^, Mohammad O. Aziz-Zanjani^1,2,3^, Mattia Dalsass^4^, Sheikh B. U. D. Farooqee^1^, Yingzhou Lu^1^, Peter K. Jackson^1,2,3^, and Capucine Van Rechem^1,5,*^.

^1^ Department of Pathology, Stanford University, Stanford, CA, USA

^2^ Department of Microbiology & Immunology, Stanford University, Stanford, CA, USA

^3^ Baxter Laboratory for Stem Cell Biology, Stanford University, Stanford, CA, USA

^4^ Immagina Biotechnology S.r.l., Pergine Valsugana (TN), Italy

^5^ Lead contact

**Supplemental figures S1 to S4**

**Figure S1.** **Related to Figure 1.** **Re-expression of SMARCB1 in rhabdoid tumor cells does not alter sensitivity to the mTOR inhibitor AZD8055.** **A.** Western blot of G401-SMARCB1 after 12h and 24h of 0.1 μg/ml of doxycycline (+) or DMSO (-) treatment in comparison to HAP1 (near haploid derived from leukemia), ReN (neuroprecursor), HEK293T (embryonic kidney).  **B-E.** Western blot of G401-SMARCB1 (**B**), TTC549-SMARCB1 (**C**), BT12-SMARCB1 (**D**), and BT16-SMARCB1 (**E**) after 24h of 0.1 μg/ml of doxycycline (+) or DMSO (-) treatment. **F-I.** Cell viability assays in the presence of the translation mTOR inhibitor AZD8055 at indicated concentrations for 72h (CellTiter-Glo 2.0 Cell Viability Assay, Promega) in G401 (**E**), TTC549 (**F**), BT12 (**G**) and BT16 (**H**) after 24h of doxycycline induction of SMARCB1 (red) or GFP (black). Cell viability was calculated as a fraction of the control (DMSO = 0 nM) viability. Averages of at least three experiments are represented.

**Figure S2. Related to Figure 2. Cytoplasmic SMARCB1 interacts with other mSWI/SNF subunits and with the translation machinery. A.** Proteins identified by mass spectrometry after immunoprecipitation of SMARCB1 from G401 cytoplasmic fraction in 300mM salt. Fold change in comparison to no SMARCB1. Green dots = mSWI/SNF subunits, magenta dots = translation-related proteins. **B.** Fold change enrichment and adjusted p values of mSWI/SNF subunits and translation-related proteins identified after SMARCB1 pull down in G401 induced with doxycycline in comparison to DMSO (left columns) and after SMARCB1 or IgG pull down in G401 induced with doxycycline (right columns). Statistics: p-values were calculated and adjusted with the Benjamini-Hochberg method, < 0.05 was considered significant. C-D. Protein-protein interaction networks for mSWI/SNF subunits (C) and translation-related proteins (D); networks performed with STRING (24).

**Figure S3. Related to Figure 3. Re-expression of SMARCB1 alters the translation efficiency of specific mRNAs. A.** Representative global translation assay in G401 cells expressing SMARCB1 or GFP after 24h induction with doxycycline. The bottom graph represents the quantification of AHA relative to GAPDH. The average of six independent experiments are represented. Statistics: * two-tailed students’ t-test, p < 0.05. **B.** Transcripts presenting alterations in ribosome occupancy (Ribo-Seq) and/or RNA levels (RNA-seq) after 12h of doxycycline induction of SMARCB1 compared to DMSO. Blue = alterations of ribosome occupancy and RNA levels in the same direction, green = alteration of ribosome occupancy, purple = alteration of RNA levels, orange = alteration of ribosome occupancy and RNA levels in opposite direction. **C.** Translation efficiency analyses from ribosome profiling and total RNA sequencing of G401 cells after 12h of doxycycline induction of GFP compared to DMSO. Significant transcripts (adjusted p value < 0.05) with a fold change > 2 are represented in red. **D.** Gene ontology analyses of transcripts presenting an increased translation efficiency upon SMARCB1 re-expression. **E.** Coding sequence length of all transcripts in the genome (green), transcripts with decreased translation efficiency upon SMARCB1 re-expression (blue), and of a random set of transcripts with comparable length of the green transcripts for further analyses (purple). **F.** GC content of transcripts with decreased translation efficiency upon SMARCB1 re-expression (blue) compared to a random set of transcripts of equal length (purple). Statistics: Mann-Whitney U test was applied for two groups and p-values were calculated and adjusted with the Benjamini-Hochberg method. *** adjusted p values < 0.001.

**Figure S4. Related to Figure 4. Re-expression of SMARCB1 increases the cytoplasmic localization of transcripts presenting increased translation efficiency. A-B.** Controls of fractionations. Relative abundance of *MALAT1* (RNA with enriched presence in the nucleus, **A**) and *S14* (RNA with enriched presence in the cytoplasm, **B**) in the nucleus and the cytoplasm in G401 with and without induction of SMARCB1 or GFP. **C-O.** Fold change of annotated transcripts in the nucleus and the cytoplasm after 24h of GFP induction with doxycycline compared to DMSO, relative to 18S. Full bars are transcripts with increased translation efficiency upon SMARCB1 induction, dotted bars are transcripts from the random set of transcripts of similar length, stripped bars are transcripts with decreased translation efficiency upon SMARCB1 induction. **P-R**. Fold change of annotated transcripts in 80S, early polysomes (EP), and late polysomes (LP) after 24h of SMARCB1 induction with doxycycline compared to DMSO. Blue are transcripts with decreased translation efficiency upon SMARCB1 induction, red with increased translation efficiency, and black GAPDH used as a control. Statistics: Student’s t test: * p<0.05 related to no SMARCB1.
