# Supplementary material for: Absence of SMARCB1 in rhabdoid tumor cells increases sensitivity to translation inhibition and alters translation efficiency of specific mRNAs
Source: J Biol Chem. 2024 Nov 13;300(12):107988. doi: 10.1016/j.jbc.2024.107988 (PMC11699736; doi:10.1016/j.jbc.2024.107988)
Supplement: Supplemental Tables [file mmc2.docx]

**Absence of SMARCB1 in rhabdoid tumor cells increases sensitivity to translation inhibition and alters translation efficiency of specific mRNAs.**

Linh T. Nguyen^1^, Anastasia E. Hains^1^, Mohammad O. Aziz-Zanjani^1,2,3^, Mattia Dalsass^4^, Sheikh B. U. D. Farooqee^1^, Yingzhou Lu^1^, Peter K. Jackson^1,2,3^, and Capucine Van Rechem^1,5,*^.

^1^ Department of Pathology, Stanford University, Stanford, CA, USA

^2^ Department of Microbiology & Immunology, Stanford University, Stanford, CA, USA

^3^ Baxter Laboratory for Stem Cell Biology, Stanford University, Stanford, CA, USA

^4^ Immagina Biotechnology S.r.l., Pergine Valsugana (TN), Italy

^5^ Lead contact

**Supplemental table 1**

| Target | Forward primer | Reverse primer |
| --- | --- | --- |
| C2orf72 | GAAGCTCAGCTCAGGAAGAC | ATCACAGTCTCCATTGGGAAATA |
| GLTPD | GCTGCTGCTGCTTTATCTCAGT | TCCTGCCGGACCTGGAA |
| FSTL3 | TGACACCGCCTGGTCCAACCT | CACGCCGTCGCACGAATCTTT |
| GATA6 | GCCACTACCTGTGCAACGCCT | CAATCCAAGCCGCCGTGATGAA |
| ATOH8 | AGCCTTCGAGGCGCTCAGGAA | TCGGCACTGTAGTCAAGGTCAG |
| CHST2 | TTTTGTGGGACTGTTGGTGA | CACCTGTTTGATCTGCTGGA |
| FOXA1 | CGCTTCGCACAGGGCTGGAT | TGCTGACCGGGACGGAGGAG |
| GFPT1 | CCCTCTGTTGATTGGTGTACGG | GGAAAAGGCAGGTTGTGCTGTC |
| IFNAI1 | GCCTTGCTGAAGTGTGGAGGAA | ATCCAGGCGATAGGCAGAGATC |
| MALAT1 | GAAGGAAGGAGCGCTAACGA | TACCAACCACTCGCTTTCCC |
| S14 | GTGTCTGCCATATCTTTGCATCC | GGTGAGGATTCATCTCGGTCTG |
| 18S | GAGGGAGCCTGAGAAACGG | GTCGGGAGTGGGTAATTTGC |
| KBTBD7 | AGTCTCTGGTGCCAGTGCCAAA | CCATCTCCTTGGCACACATACC |
| CCR1 | CAACTCCGTGCCAGAAGGTGAA | GTTCAGGAGGTAGATGCTGGTC |
| ANGPT2 | ATTCAGCGACGTGAGGATGGCA | GCACATAGCGTTGCTGATTAGTC |
| SKAP1 | CAGCCAGATGAACTGTCCTTCC | GGAACAATCCCAACGAGGCTGT |
| GAPDH | CCACCCATGGCAAATTCC | TGACAAGCTTCCCGTTCTCA |

**Table S1. List of primers.**
